# “Now I Feel That I Can Achieve Something”: Young Tanzanian Women’s Experiences of Empowerment by Participating in Health Promotion Campaigns

**DOI:** 10.3390/ijerph18168747

**Published:** 2021-08-19

**Authors:** Ingrid Espegren Dalsmo, Kristin Haraldstad, Berit Johannessen, Olav Johannes Hovland, Mercy G. Chiduo, Liv Fegran

**Affiliations:** 1 Department of Health and Nursing Science, Faculty of Health and Sport Sciences, University in Agder, 4604 Kristiansand, Norway; kristin.haraldstad@uia.no (K.H.); berit.johannessen@uia.no (B.J.); johannes.hovland@uia.no (O.J.H.); liv.fegran@uia.no (L.F.); 2 National Institute for Medical Research (NIMR), Tanga Centre, Tanga 21101, Tanzania; mercy.chiduo@nimr.or.tz

**Keywords:** health promotion, campaign, youth program, dropout, young women, empowerment, Tanga International Competence Centre (TICC), Tanzania

## Abstract

The United Nations (UN) emphasizes that health promotion, education, and empowerment of women are all goals that will help to end poverty. In eastern rural Tanzania, young women who dropped out of school now take an active part in health promotion campaigns in schools and villages through the youth program “Innovative and Productive Youth”, which is administered by the nongovernmental organization Hatua na Maendeleo (HAMA). The aim of this qualitative study was to explore how some of these young Tanzanian women experience participating in health promotion campaigns. A hermeneutic phenomenology design with focus group interviews was used. The study’s participants were nine young women between the ages of 18 and 23 who had participated in the youth program for one year. In addition, the participants were given the opportunity to provide written elaboration in Kiswahili after the interviews. The findings were analyzed from an empowerment perspective and revealed the benefits that the young women had experienced, which were expressed as three themes, i.e., my involvement in the campaigns (a) made me strong and confident, (b) made me become a role model, and (c) made me think that I can achieve something. Involvement in health promotion campaigns seemed to empower the young women by increasing their confidence and providing a feeling of self-efficacy. In addition, their health literacy increased, which appeared to have a ripple effect on their families, peers, and the local community. The findings from this study provide insight into the participants’ self-reported short-term effects. Moreover, with this study, it can be argued that by empowering individuals, community transformation can be seen as well.

## 1. Introduction

The United Nations’ (UN) 2030 Agenda for Sustainable Development [1] states that poverty in all its forms and dimensions is the greatest global challenge and identifies health promotion, education, and empowerment of women as goals that will help to end poverty [1]. The Tanzania Human Development Report 2020 shows that levels of poverty in Tanzania are high, as assessed by indicators of development [2]. Empowerment is one of the main goals of the Tanzanian nongovernmental organization “Hatua na Maendeleo” (HAMA), which means “steps for development.” HAMA collaborates with local and regional authorities in rural Tanzania to achieve the UN sustainability goals via seven community development programs that are supported by its parental organization, the Tanga International Competence Centre (TICC) [3]. TICC works to achieve competence and community development by connecting people across borders, combining knowledge and experience, and co-creating a new model for regional development [3].

The HAMA community development programs use positive psychology (appreciative inquiry, AI) to focus on developing people’s strengths [4]. AI is an approach to personal change that assumes that questions and dialogue about strengths, successes, values, hopes, and dreams are themselves transformational. Through inquiry and dialogue, people can shift their attention and actions away from problem analysis to identifying worthy ideals and possibilities for the future [4]. Furthermore, HAMA is based on the philosophy that people will feel empowered when they see a connection between their efforts and their life outcomes [3].

### 1.1. Youth Program and Health Promotion Campaigns

One of HAMA’s programs, “Innovative and Productive Youth,” targets school dropouts. Dropping out of school or not starting school at all is a major problem in Tanzania. In 2018, 56% of children of secondary school age (14–19 years) were not enrolled in school [5]. The participants in the youth program are recruited from the rural villages in close collaboration with the local leaders. The young participants receive tuition to allow them to catch up with the academic knowledge they have missed by not attending school, together with a range of self-development classes in areas such as awareness and culture. They also take part in the other HAMA community development programs that target different age groups, such as the Health Campaigns Program. The goal of the youth program is for participants to finish secondary school or start vocational training after one year in the program.

The Health Campaigns Program aims to increase the community’s health knowledge by incorporating dance, music, role-play, and quizzes into the campaigns, so to educate the community in both the schools and villages. Examples of health issues addressed are the importance of drinking enough water, daily life hygiene, prevention of malaria, nutrition, and mental health issues.

### 1.2. Empowerment

Since the Ottawa Charter for Health Promotion [6], empowerment has been one of the core principles of the World Health Organization’s (WHO) approach to health promotion. Empowerment relates to coping with challenges and overcoming a sense of powerlessness, and it is an important part of public health practice [7]. Empowerment can be seen as both a process and a result and has no unambiguous definition [8]. Individual empowerment, which is the focus of this study, refers primarily to a person’s ability to make decisions and have control and relates to individual characteristics such as general self-efficacy [9,10].

**Figure 1:**
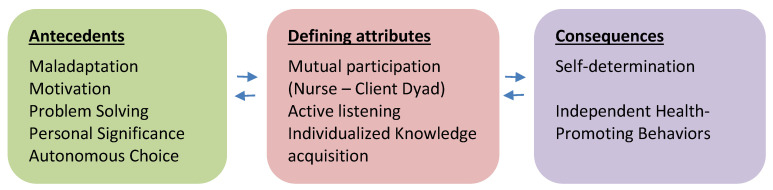
Ellis-Stoll and Popkess-Vawter’s model of the empowerment process.

Several concept analyses of empowerment have been reported in both generic and diagnosis-specific contexts [11,12,13,14]. Ellis-Stoll and Popkess-Vawter’s model of empowerment in nursing was used as the theoretical basis for the present study [11] because it focused on empowerment as a process that can result in independent health-promoting behaviors. Ellis-Stoll and Popkess-Vawter [11] define empowerment as *“a participative process through a nurse–client dyad that is designed to assist in changing unhealthy behaviors”* [11]. Figure 1 outlines the antecedents, defining attributes, and consequences of their model of the empowerment process.

The levels of gender equality and women’s empowerment have increased in recent decades [15]. Nevertheless, this is still a global challenge, and a lack of gender equality is a major obstacle to sustainable development [15]. In most sub-Saharan countries, Tanzania included, girls are less likely than boys to complete secondary school [5,16]. Because of this gender inequality and the fact that the UN emphasizes women’s education and empowerment, this study concentrated on the young women who were involved in the youth program and its associated campaigns.

The main aim of this study was to explore how young women who dropped out of school experience being involved in health promotion campaigns. Further, we wanted to explore how their involvement in health promotion campaigns impacted their empowerment with regard to coping strategies, continued education, health awareness, and their ability to support themselves and others in health-related issues.

## 2. Methods

### 2.1. Research Design

We chose a qualitative design with a hermeneutic phenomenology approach to explore the participants’ experiences of being involved in health promotion campaigns [17]. In a hermeneutic phenomenology approach, the researcher’s perceptions about the study’s topic should be transparent, and the researcher must use a reflective attitude and approach throughout the process [17]. In this study, the data were collected by the first author, who is a nurse currently working as a nurse teacher at a university in Norway that collaborates with the TICC and HAMA. This is how the first author became interested in the youth program and the health promotion campaigns in which the youth participated.

### 2.2. Study Context

The study was focused on and conducted in rural Tanzania in the setting of the HAMA organization and was conducted at the premises of the TICC, at primary schools, and in villages in a rural district in Tanzania where the campaigns were conducted.

### 2.3. Study Participants

A purposive sampling of all of the nine young women in the youth program at the time of data collection was chosen. The inclusion criteria were that the participants had dropped out of school early and that they had participated at least once in health promotion campaigns. To improve understanding of the health promotion campaigns, the first author accompanied the young women to campaign activities before and after the focus group interviews.

### 2.4. Data Collection

Data were collected through two focus group interviews with four and five participants in each group. In addition, the participants were given the opportunity to complement the interview data in writing after the focus group interviews. We established a collaboration with a senior research scientist at the National Institute of Medical Research, Tanga Medical Research Centre (NIMR), prior to the data collection. The staff at the TICC assisted with translating the consent form and interview guide. During the focus group interviews, a Tanzanian health worker from the TICC/HAMA interpreted orally from English to Kiswahili and vice versa so that the participants could speak in their native language.

### 2.5. Focus Group Interviews and Written Answers

Focus groups interviews were chosen as the investigative method because the research topic dealt with common experiences, attitudes, and views of a group of young people, all of whom were involved in the same health promotion campaigns. Focus group interviews can contribute to associations and group dynamics that provide examples and stories that may highlight the theme [18]. The participants were familiar with each other, and therefore, they were more willing to discuss the topic with their peers as opposed to being interviewed individually. A semi-structured interview guide was used to provide a framework of themes to be explored, e.g., the overall experience of participation in the campaigns, health-related knowledge, and continuance of education.

After the focus groups, the participants were given the opportunity to provide written elaboration in Kiswahili of issues that were discussed during the interviews. This was conducted to ensure that the use of an interpreter did not restrain the participants’ descriptions; however, no new information emerged from these additional written answers. The interviews were audio-recorded and transcribed verbatim by the first author.

### 2.6. Data Analysis

All text from the data collection was imported to the NVivo 11 software program (by QSR International) for further organization and analysis [19]. We performed a qualitative content analysis using a hermeneutic phenomenology approach [17]. In the first phase, a naïve reading of the transcribed interviews was performed in order to obtain a first impression of the text as a whole in relation to the young women’s experience of participating in health promotion campaigns. In the second phase, structural analyses helped divide the text into units of meaning that were condensed, abstracted, and structured into themes. A comprehensive understanding was developed in the third phase, where the authors’ preconceptions, the naïve reading, the structural analyses, and the relevant literature were all taken into account.

## 3. Findings

All nine individuals aged 18–23 years who met the inclusion criteria were invited and agreed to participate in the study. The findings were related to empowerment based on the benefits that the young women reported, and three main themes emerged: the involvement in the campaigns (a) made me strong and confident, (b) made me become a role model, and (c) made me think that I can achieve something.

### 3.1. Made Me Strong and Confident

One of the most important findings was that the participants experienced increased confidence; eight of nine participants expressed this clearly. They highlighted that they underwent a personal change while being involved in the campaigns as a part of the youth program. The most common change was a transition from being shy to becoming confident. One participant noted, “*I am one of those who has benefitted. Before, even at school, I was very shy. I couldn’t even stand in front of the other pupils to say anything. (…) Now I can stand in any community and talk about health* (P3).”

Many of the young women noted that this happened not only in the campaigns but in other aspects of their daily life as well. One participant noted, “*And if it is, for example, like a political position, I can stand up in front of people and fight for it* (P2).”

Confidence also increased the feeling of self-efficacy. One of the young women described how increased confidence enabled her to make decisions in her own life (P5). The crucial role of the young women in the campaigns clearly gave them a feeling of importance and self-worth. One participant explained, *“I’m proud because I have learned a lot through the campaigns, and I have trained a lot of people… So now I’m proud of myself* (P6).”

All the participants described how they learned about different health topics from the campaigns. They discussed what they learned and stressed the importance of, e.g., drinking water, how to prevent themselves from getting malaria, personal hygiene, and protection against HIV/AIDS. This clearly showed that both the audience for the campaigns and the young participants themselves gained health knowledge. One participant said, ”*Yes, it has enhanced my awareness and expanded my understanding of what is right and what is wrong* (P1).”

### 3.2. Made Me Become a Role Model

Two of the young women talked about the importance of setting a good example when they educate others on health topics (P3 and P5). Most of the others agreed and considered it deceptive to not act as good role models for health promotion. One participant noted, “*Because the campaigns start with you. What you learn from the campaign starts with you. So, if I go to tell someone the importance of drinking enough water, I have to be a mirror. You have to let them learn from you* (P5).”

The participants emphasized that their own health behaviors had changed during the previous year and that teaching others had been crucial in this process. The young women gave several examples of how they used their new knowledge to change their habits. One participant stated, “*I have gained knowledge from the campaign about drinking enough water. I used not to drink water, but after the campaign, I understood its benefits. Nowadays I drink enough water and I feel that my body is healthy* (P4).”

Moreover, most of the participants expressed that they wanted to remember what they learned so as to use it in the future. One participant stated, “*I’ll make sure that what I have learned from the campaigns, I’ll practice. I’ll use it for my child. For example, eating and washing at the right time, and using all the other things that are needed for my baby (P9).”*

### 3.3. Made Me Think That I Can Achieve Something

All the participants noted the importance of being able to manage their own lives and support their families. The young women stated that they could now envision a future where they would be able to give something back to those around them. They clearly felt a responsibility for themselves, as well as their family and friends. One participant commented, “*After this I will start vocational training and I will manage to work and support myself and my family* (P8).”

The participants spoke about their future in a positive way. Several of the young women clearly expressed that being in the youth program had helped them to gain a more positive attitude toward their education. Some had planned a specific career and reported a renewed motivation to continue their education. Five of the participants were determined to work as health workers or health educators or to volunteer in health promotion work in the future. While some had new careers planned, others had no specific plans but described a renewed hope for the future because of their increased self-confidence. One participant stated, “*Yes, I feel that I can do anything. I do believe in myself now* (P7).”

When the young women were involved in campaign work in the evenings, they were paid by HAMA. The participants reported that this helped them financially by enabling them to be more economically independent. It also had positive effects on their future outcomes. One participant noted, “*The income I earn can be saved and pay for my education* (P1).”

## 4. Discussion

This study provided new insight into the experiences of young women who had dropped out of school and were involved in health promotion campaigns in eastern rural Tanzania. Our findings will be discussed in light of the empowerment process described by Ellis-Stoll and Popkess-Vawter [11].

### 4.1. Antecedents of Empowerment

For these young women, the antecedents required for beginning an empowerment process were clearly present in the Innovative and Productive Youth program [11]. First, the participants expressed that their health behaviors were poor prior to their involvement in the health promotion campaigns. This experience can be related to the concept of maladaptation in the Ellis-Stoll and Popkess-Vawter model, which can manifest itself in poor health behaviors [11]. By teaching others about different health issues during the campaigns, the young women at the same time increased their own health knowledge.

Motivation is a crucial part of starting the empowerment process. The participants had autonomously made the decision to join the youth program and were motivated to change their life situations. Their involvement in the campaigns was clearly of personal significance for them. Their statements about being able to achieve something and having renewed hope for their future indicate that involvement in the campaigns increased their motivation. These findings are consistent with those of studies that describe the importance of motivation to implement desirable behavioral change [20,21].

The ability to problemsolving is necessary to achieve the goal of changing one’s situation [11]. The participants described how the youth program and health promotion campaigns made them change their own health habits, in addition to finishing their education or starting vocational training. These findings relate to those of earlier research on empowerment, which identified problem solving as an important aspect [22]. Involving young women in the youth program may have contributed to solving both the community problem concerning dropouts and improving its low level of health knowledge among the residents. This relates to theories that the empowerment of individuals can also have an impact on the community [6,7,10,14].

### 4.2. Defining Attributes of Empowerment

The defining attributes of empowerment, as described by Ellis-Stoll and Popkess-Vawter [11], were present for the young women in the youth program. An important finding was that involvement in the program and the campaigns led participants to feel both strong and confident. This might be because of the important role the participants played in the campaigns and the high degree of mutual participation between the young women themselves and between the HAMA employees and the young women. Mutual participation is crucial for learning and the establishment of goals [11]. It is especially valuable for empowering marginalized youth [23]. Empowerment can also be seen as a transactional process that involves relationships with others and encourages the sharing of resources and collaboration [13]. Collaboration, mutual participation, and a sense of connection are all important aspects of the empowerment of young people [22,24,25].

The method of AI used in the HAMA projects requires active listening, which is one of the defining attributes of the empowerment process [11] and a prerequisite for knowledge acquisition to occur [4,11]. The opportunity-centric methodology used in AI looks at the change in people through positive “glasses” [4] by focusing on young people’s strengths, successes, values, hopes, and dreams [3]. This can be linked to empowerment as a developmental process, whereby growth and potential are enhanced [13]. During the campaigns, the young women mastered performance through exceptional acting, presenting, singing, and dancing; this affirmed HAMA’s focus on the young women’s strengths and abilities. AI appears to have positive consequences for integrating youth development and community building, as described by Nitzberg [24]. Further, our findings clearly highlighted that all the participants had experienced increased health literacy through their participation in the campaigns. In the group interviews, the young women gave many examples of issues they learned about and were able to transfer to their own lives. Health literacy is regarded as a personal resource that evolves over the course of one’s life and promotes empowerment in health decision-making [26]. Consistent with their growing empowerment, the young women reported acquisition of individualized knowledge [11]. In this context, empowerment particularly focuses on an individual’s ability to have control over what affects their health. Greater access to resources such as knowledge of the importance of developing critical awareness has been reported to be a key factor in empowerment [13,14].

### 4.3. Consequences of the Empowerment Process

Consequences relate to outcomes of the empowerment process, and the findings of this study demonstrated the positive outcomes, for both individual and community, of participation in the youth program and the health promotion campaigns. The three main themes identified from the young women’s experiences, i.e., “made me strong and confident,” “made me become a role model,” and “made me think that I can achieve something,” can all be seen as positive outcomes.

Independent health-promoting behaviors were described as developing from personal knowledge and empowerment that could be expanded to support others such as family and friends in health-related issues [11]. The outcomes of increased self-confidence reported by the young women in this study coincide with the health promotion outcomes described by WHO, i.e., promoting people as the main health resource and highlighting the process of enabling people to improve their own health [8,27].

Many of the concept analyses of empowerment emphasize a feeling of hope as one of the consequences of empowerment [12,13,15]. The participants clearly described how going through an empowerment process gave them a feeling of self-efficacy and renewed hope for the future. These findings are consistent with those of earlier studies that reported increased confidence and personal strength, as well as a sense of mastery and control in those who experienced increased empowerment [22,28,29,30]. The young women’s hopes for the future can be seen as a consequence of empowerment because their self-determination increased [11]: they were now at a different place in life than they would have been without their involvement in the youth program and health promotion campaigns.

### 4.4. Methodological Considerations

The focus group methodology was chosen to allow the young women to reflect on their mutual experiences of being involved in health promotion campaigns.

The use of an interpreter could be a limitation [31]. To compensate, the researcher and interpreter clarified the focus of the group interviews before the data collection. To quality check the interview translation, a Tanzanian senior research scientist at NIMR compared the audio recordings with the transcribed text before the analysis.

The sample was small, but all eligible participants were asked and consented to participate. Ideally, focus groups should have six to ten participants in order to facilitate a dynamic conversation between the group members [32]. Thus, the group size in this study was slightly below that recommended [32]. However, the participants provided rich data as they had experience from multiple campaigns. The new knowledge provided orally and in writing by the participants adds in-depth insight to understanding their involvement in health promotion work, implying information-power [33]. 

The homogeneous positive feedback from the participants could be considered a weakness of the study. However, it is not surprising that most of the experiences were positive because the youth program provides an opportunity for young women to promote personal development in rural Tanzania. To ensure that all aspects of the participants’ experiences were included in the data material, an opportunity to give additional written information after the group interviews was provided. Quotes from all the participants presented in the findings section of this article substantiate our findings.

The transparent study design may make the findings transferable to similar participants in similar contexts, and hence the findings may provide valuable insight into factors of importance for empowering young women.

The authors’ positive perceptions of HAMA and their work might also have influenced the interviews, the analysis process, and/or the findings. To compensate for this bias, there was a focus on maintaining a reflective attitude by means of regular discussions with the co-authors. In addition, all the steps of the research process, from data collection and analysis to presentation of findings, have been described in detail.

## 5. Conclusions

A future in which women are healthy, educated, protected, and empowered is the goal of the UN 2030 Agenda for Sustainable Development [1]. Putting peoples’ capacity first is a key to social transformation, empowerment, and improved health outcomes. By including the young women in both the Health Campaigns Program and the Innovative and Productive Youth Program, it seems that HAMA has made a beneficial step towards the vision of strengthening the health and education of people in local Tanzanian communities. While this is a small-scale study, the youth who participated in the campaigns appear to have been empowered at the same time as the residents in the community received health information themselves. Nevertheless, the findings from this study only provide evidence on the participants’ self-reported short-term effects of participating in health promotion campaigns. Therefore, further research, guided by the Ellis-Stoll and Popkess-Vawter model of the empowerment process (Figure 1), should focus on the long-term effects of involvement in the campaign program. It would also be interesting to explore health promotion campaigns from a public health perspective.
